# Disinfection

**Published:** 1902-09-27

**Authors:** 


### Hospital Clinics and Medical Progress.

### DISINFECTION.

The more carefully we study the subject of disinfection the more difficult does it appear to devise any method on which complete reliance can be placed. Yet the methods which give moderately good results are fairly numerous, and thus it happens that although we can rarely assert with any degree of positiveness that a house and its contents have been so thoroughly disinfected as to be absolutely incapable of conveying infection, we may very often be, as the saying is, "practically" sure that all is right. Much of the difficulty of the whole problem undoubtedly hinges on the fact that all processes which are included in the term disinfection aim at producing an immediate and complete result by one operation; for if one is content to take time, and repeat the process, comparatively simple measures are often sufficient. For example it can hardly be doubted that by repeatedly rubbing down the walls and ceilings, by the repeated use of soap and hot water upon the floors, by aid of a scrubbing brush and by exposure to fresh air, a room may be restored to its pristine innocency. But that takes time, so that although such ordinary domestic disinfection may probably be quite sufficient where a room can be left unoccupied for a considerable time, or where a whole house can be handed over for cleansing purposes and left uninhabited for some weeks, it entirely fails of its purpose where it is necessary to bring the infected room into use again at short notice. In a very complete essay on "Practical Disinfection in Schools," Dr. Houston [1] enters fully into the subject—so fully in fact that we can but fear that some of his readers may come to the conclusion that the attainment of a complete disinfection is such a difficult proceeding as to be a hopeless undertaking. This, however, is far from being the case if we will be content with practical rather than theoretical completeness. After describing and discussing the various methods in use he says that, if it were necessary to summarise the main points relating to practical disinfection in the fewest possible words, without reference to alternative methods or a choice of different disinfectants, an extremely concise but reasonably safe statement would be as follows:—

For rooms and for furniture, books, boots, shoes, and articles liable to be injured by steam disinfection, formic aldehyde vapour, preferably Lingner's (glycoformal) apparatus, one apparatus for a room 2,800 cubic feet, 2 litres of glycoformal being used. Duration of exposure, four hours.

For general washing purposes, corrosive sublimate, 1 in 1,000.

For excreta, sputum, and vomited matters, corrosive sublimate, 1 to 500. Duration of contact, one hour. The mixture to be thorough.

For linen, cups, saucers, plates, and knives, boiling water.

For bedding, clothes, hangings, carpets, and under garments (other than linen), saturated steam at 115° C. for 30 minutes.

In regard to room disinfection it is to be noted that there is a decided advantage in combining gaseous disinfection with spraying or washing of exposed surfaces. But in ordinary cases of infectious disease (but not in tubercle infected houses) gaseous disinfection with formalin vapour and washing painted or varnished surfaces and scrubbing the floor with disinfectant solution (sublimate, 1 in 1,000; carbolic acid, 1 in 50; or izal, 1 per cent.) may be considered sufficient. In the disinfection of the excreta, especially the stools, there is much to be said in favour of heat. The difficulties are the smell, which, however, can be obviated by the addition of potassium permanganate and the disagreeableness of the process. The latter in an institution would of course be got over by the use of a special apparatus; but in individual cases it is worth noting that the destruction of the bacilli, and not necessarily of the resistent spores of bacteria, will afford reasonable safety; so that the addition of a considerable excess (four times the bulk of the substance to be disinfected) of boiling water will often do all that is absolutely necessary. Thus as an alternative to the use of sublimate about two teaspoonfuls of the crystals of potassium permanganate may be added to two pints of water in an enamelled vessel, brought to the boil, and at once poured over the stool. There is one advantage in this process over that with corrosive sublimate, namely, that the disagreeable work of thorough mixing is not so necessary. The disinfection of linen seems at first sight a simple matter, and the ordinary householder solves the problem by sending it to the wash. But that is not enough. One has to bear in mind on the one hand that infected linen must be rendered innocuous before it is sent to the laundry, and on the other that linen that has been soiled by various discharges cannot be at once placed in boiling water without fixing these discharges in the fabric and thus permanently injuring it. The following alternative plans, therefore, are recommended:—(1) Steep the linen for an hour in a relatively large bulk of a solution of corrosive sublimate 1 : 1,000, or izal 1 : 100, wring out, and send to the "wash" in the moist condition. (2) Steep the linen for an hour in cold water contained in a caldron capable of being heated. Bring the water by heating to a temperature of 100° C. After 10 minutes, or when sufficiently cool to handle, wring out the linen and send it to the "wash" without preliminary drying. In neither case should the linen be "washed" on the infected premises.

[1] Practitioner, September.

